# Preliminary study on the expression of endothelial cell biology related genes in the liver of dengue virus infected mice treated with *Carica papaya* leaf juice

**DOI:** 10.1186/s13104-019-4242-z

**Published:** 2019-04-03

**Authors:** Mohd Ridzuan Mohd Abd Razak, Nor Azrina Norahmad, Nur Hana Md Jelas, Bazilah Jusoh, Amirrudin Muhammad, Norazlan Mohmad Misnan, Murizal Zainol, Ravindran Thayan, Ami Fazlin Syed Mohamed

**Affiliations:** 1 Herbal Medicine Research Center, Institute for Medical Research, Kuala Lumpur, Malaysia; 2 Infectious Disease Research Center, Institute for Medical Research, Kuala Lumpur, Malaysia

**Keywords:** Gene expression, Virus, AG129 mice, Endothelial cell biology, Dengue, Plants, qRT-PCR, *Carica papaya*, Liver, In vivo

## Abstract

**Objective:**

The purpose of this study was to profile and identify the endothelial cell biology related genes that are affected by dengue virus infection in the liver tissue of AG129 mice, with and without *Carica papaya* leaf juice treatment.

**Results:**

The dengue fever mouse model was established by intraperitoneal inoculation of dengue virus, New Guinea C strain at 2 × 10^6^ PFU. Daily oral administration of 1000 mg/kg freeze-dried *C. papaya* leaf juice (FCPLJ) was done starting from day 1 to day 3 post infection. The RNA was extracted from liver tissues harvested on day 4 post infection. The expression levels of 84 genes related to mouse endothelial cell biology were determined by qRT-PCR technique. Dengue virus infection upregulated 15 genes and downregulated two genes in the liver of AG129 mice. The FCPLJ treatment upregulated monocyte chemoattractant protein 1 and downregulated intercellular adhesion molecule 1, integrin beta 3 and fibronectin 1 genes during dengue virus infection. The data showed the potential effect of FCPLJ treatment on the expression profile of endothelial cell biology related genes in the liver of dengue virus infected-AG129 mice. Further proteomic studies are needed to determine the functional roles of the genes affected by FCPLJ treatment.

**Electronic supplementary material:**

The online version of this article (10.1186/s13104-019-4242-z) contains supplementary material, which is available to authorized users.

## Introduction

The transport of molecules across the blood vessels or endothelial permeability provides nutrients to the underlying tissues and supports several physiological functions such as leukocyte transmigration and hemostasis [1]. In pathological conditions, endothelial dysregulations or a change in endothelial functions toward reduced vasodilation, pro-inflammatory and pro-thrombic states have been associated with cardiovascular diseases, diabetes, chronic kidney failure and severe viral infections [2]. In dengue virus infection, the increase in endothelial permeability was caused by the interaction of immune mediated inflammatory molecules produced by the host in response to the infection [3]. The liver, which is surrounded by sinusoidal endothelium [4], has been shown to be the target of dengue virus based on the increase level of liver function markers [5], the abnormal histopathological changes and the appearance of dengue viral antigen in the liver tissue [6–8]. However, the molecular evidence of endothelial permeability regulation during dengue virus infection in the liver is lacking. As endothelial permeability could be enhanced by the interaction of inflammatory molecules at the site of infection [3], any potential immunomodulators that could stop this interaction may also prevent the progress of severe dengue infection. Apart from anti-thrombocytopenic activity, the *Carica papaya* leaf juice has been proven for its immunomodulatory activities in vitro [9] and in vivo [10, 11]. However, the potential of *C. papaya* leaf juice in affecting the regulation of endothelial permeability during dengue virus infection is unknown. Therefore, identification of endothelial cell biology related genes in the liver of dengue virus-infected AG129 mice that are affected by *C. papaya* leaf juice treatment may provide initial evidence to support future studies focusing on the role of the juice in endothelial permeability regulation during dengue virus infection.

## Main text

### Methods

#### Ethical statement, animal husbandry and study design

The experiments and procedures involving study animals were approved by the Animal Care and Use Committee, Ministry of Health Malaysia, (ACUC/KKM/02(9/2016)). The study animals, AG129 mice (alpha/beta and gamma interferon receptors knockout 129/Sv mice) (males, 7–8 weeks old) were purchased from Marshall BioResources, United Kingdom. The in vivo experiments were performed in the Non-clinical Research Facility, Laboratory Animal Research Unit, Medical Research Resource Center, Institute for Medical Research, Kuala Lumpur, Malaysia. The mice were housed in individual ventilated cages supplied with corn cob beddings. The reverse osmosis drinking water and mouse pellet were supplied ad libitum. The mice were exposed with artificial light to mimic the 12 h light and 12 h dark. The room temperature ranged between 20 and 26 °C. The acclimatization was done for 7 days prior to the experiment. The morbidity level was monitored once a day based on the method described previously [12].

The mice were assigned into 4 experimental groups by simple randomization technique. The first group was the mock infected mice (n = 3), which were inoculated intraperitoneally with 0.2 ml sterile plain media. The second group was the infected mice (n = 3), which were inoculated with 2 × 10^6^ PFU of non-adapted serotype 2 dengue virus New Guinea C strain (ATCC VR-1584) as mentioned in a previous study [13]. The third group was the infected mice with treatment (n = 4). The last group was the mock infected mice with treatment (n = 3).

#### Freeze-dried C. papaya leaf juice (FCPLJ) preparation and dosing

The fresh green leaves were harvested from the *C. papaya* trees (‘Sekaki’ cultivar) grown organically in the herbal garden of the Institute for Medical Research, Kuala Lumpur, Malaysia. A voucher specimen (Voucher No: 007/10) was prepared by Ms. Tan Ai Lee and archived in the Forest Research Institute Malaysia, Kepong, Malaysia. The FCPLJ was prepared as described in a previous study [13]. The FCPLJ has been phytochemically characterized previously. At least 3 compound groups consisting of phenolic acids, piperidine alkaloids and glycosylated flavanols were detected [13, 14].

The dose for this study, 1000 mg/kg BW, was chosen based on the non-toxic dosing range used in the previous toxicology studies of FCPLJ [14–16]. The dose was also two times higher than the human equivalent therapeutic dose used in the previous clinical trial of CPLJ [13, 17]. The dosing volume was based on the 10 ml/kg bodyweight requirement. The dosing was done orally, once a day for 3 consecutive days (Day 1, Day 2 and Day 3 post infection).

#### Liver organ harvesting and RNA extraction

The mice were euthanized by open-drop exposure to isoflurane on Day 4 post infection. The livers were harvested and kept in RNAlater^®^ solution (Thermo Fischer Scientific, USA) and stored at − 40 °C prior to RNA extraction. Approximately 30 mg of the liver tissues were homogenized using TissueRuptor^®^ (Qiagen, USA). The total RNA was extracted using RNeasy mini kit (Qiagen, USA) as per manufacturer’s instructions. The concentration and purity of the extracted RNA was determined by using NanoDrop spectrophotometer (Thermo Scientific, USA).

#### Quantitative reverse transcription PCR (qRT-PCR)

The complementary DNA (cDNA) was synthesized from 1500 ng of total RNA using QIAGEN first-strand kit as per manufacturer’s instructions. The synthesized cDNA samples were stored at − 20 °C for later use. The qRT-PCR was performed by using the Qiagen mouse endothelial cell biology RT^2^ Profiler PCR array (PAMM-015Z) in combination with RT^2^ SYBR^®^ Green qPCR Mastermix (Qiagen, USA) as per manufacturer’s recommendations. The viral RNA level was quantitated by qRT-PCR (Applied Biosystems 7500 fast, USA) as mentioned in a previous study [13].

#### Data analysis

The cycle threshold (Ct) values were normalized based on a combination of five housekeeping genes (Beta actin, beta-2 microglobulin, glyceraldehyde-3-phosphate dehydrogenase, heat shock protein 90 alpha class B member 1 and beta glucuronidase). The fold regulation and statistical analysis were calculated by the data analysis web portal (http://www.qiagen.com/geneglobe). The p values were calculated based on a Student’s t-test of the replicate 2^(−∆ Ct)^ values for each gene.

### Results

#### Expression of genes related to endothelial cell biology in the liver of dengue virus infected-AG129 mice

**Fig. 1:**
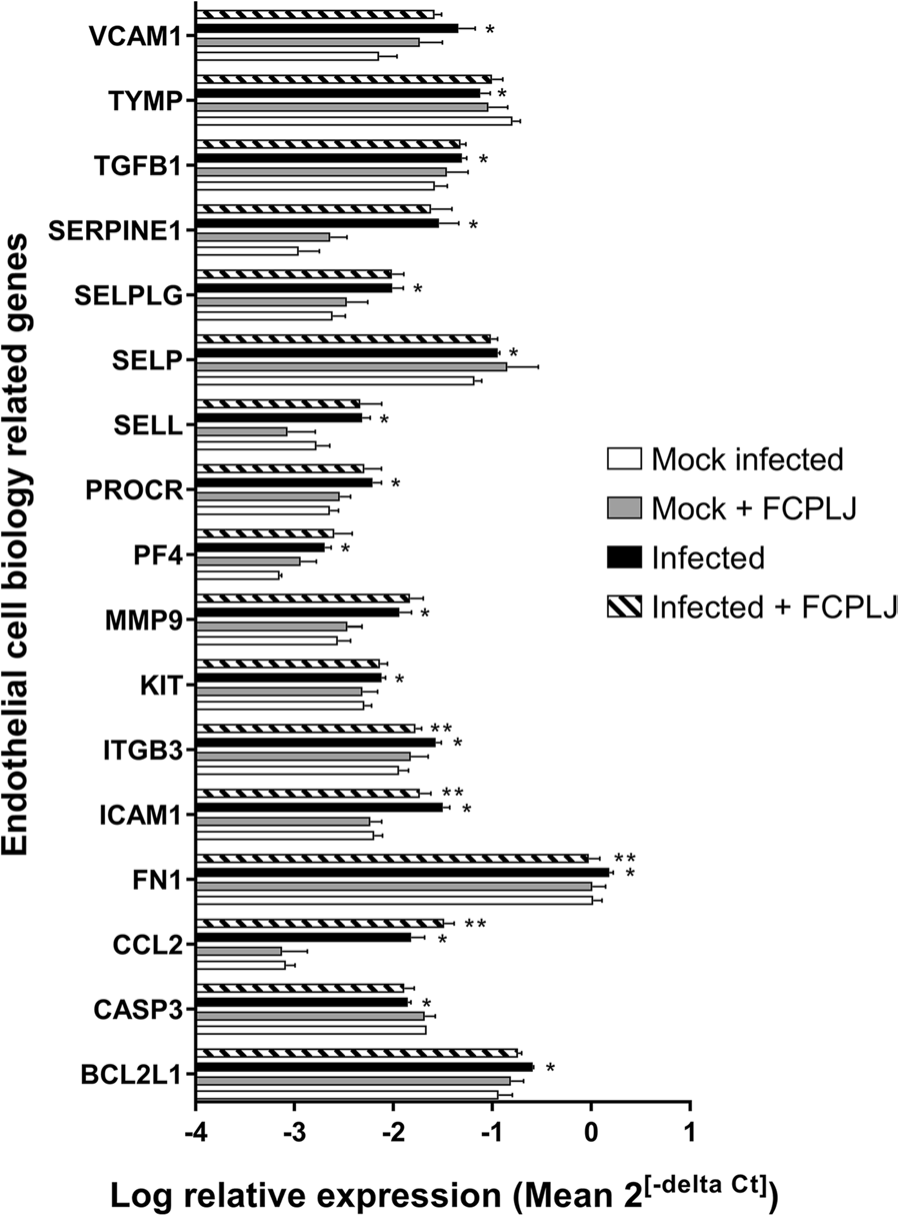
The endothelial cell biology genes affected by dengue virus infection in the liver of AG129 mice. The gene expression analysis was done using the liver RNA sample of mock infected (white bar) (n = 3), mock infected + FCPLJ (grey bar) (n = 3), infected (black bar) (n = 3) and infected + FCPLJ (lined bar) (n = 4) mice. Seventeen genes were affected by dengue virus infection (as compared to mock infected). Treatment with FCPLJ affected 4 genes in the liver of dengue virus infected AG129 mice. Each bar represents the log mean relative expression value ± SD. *Denotes significant changes as compared to mock infected group, **denotes significant changes as compared to infected mice

The RNA samples from all mice groups, mock infected (n = 3), infected (n = 3), infected + FCPLJ (n = 4) and mock infected + FCPLJ (n = 3) were included in the analysis. The liver tissues, which were harvested on day 4 post infection, were confirmed to be infected with dengue virus by the presence of dengue viral RNA (Additional file 1). The viral RNA level in the liver of dengue virus infected AG129 mice was not affected by FCPLJ treatment (Additional file 1). The mouse endothelial cell biology RT^2^ Profiler PCR array has enabled us to analyze the expression level of 84 genes (Additional file 2) in the liver. As compared to the mock infected group, 17 out of 84 genes were significantly (*p *< 0.05) affected by dengue virus infection in the liver (Fig. 1). Among the affected genes, 15 genes were upregulated (~ 1.49- to 27.98-folds) and 2 genes were downregulated (~ 1.56- to 2.14-folds) during dengue virus infection in the liver (Table 1).

**Table 1:** Fold regulation of endothelial cell biology genes in the liver of dengue virus infected mice as compared to mock infected mice

Gene symbol	Description	Fold regulation^a^	*p* value^b^
SERPINE1	Serine (or cysteine) peptidase inhibitor, clade E, member 1	27.9817	0.046792
CCL2	Chemokine (C–C motif) ligand 2	17.9509	0.010773
VCAM1	Vascular cell adhesion molecule 1	6.4031	0.039317
ICAM1	Intercellular adhesion molecule 1	4.9573	0.001673
MMP9	Matrix metallopeptidase 9	4.2087	0.017157
SELPLG	Selectin, platelet (p-selectin) ligand	4.0692	0.012107
SELL	Selectin, lymphocyte	2.9629	0.008503
PF4	Platelet factor 4	2.8456	0.002018
PROCR	Protein C receptor, endothelial	2.6883	0.010671
ITGB3	Integrin beta 3	2.3885	0.004266
BCL2L1	Bcl2-like 1	2.3017	0.005111
TGFB1	Transforming growth factor, beta 1	1.9354	0.018087
SELP	Selectin, platelet	1.7322	0.00361
KIT	Kit oncogene	1.51	0.019643
FN1	Fibronectin 1	1.4884	0.038106
CASP3	Caspase 3	− 1.5606	0.000338
TYMP	Thymidine phosphorylase	− 2.1416	0.015268

AG129 mice were intraperitoneally inoculated with 2 × 10^6^ PFU dengue virus, New Guinea C strain. Mock infected AG129 mice were intraperitoneally inoculated with media only

^a^Fold regulation of > 1.5 and > − 1.5 were considered upregulation and downregulation, respectively as compared to the gene expression level in the mock infected mice

^b^The differences in gene expression level was considered significance at a *p* value of < 0.05

#### The effect of FCPLJ treatment on the regulation of endothelial cell biology related genes in the liver of dengue virus infected AG129 mice

In order to determine the effect of FCPLJ treatment during dengue virus infection, the gene expression data of infected AG129 mice treated with FCPLJ was compared with the gene expression data of the infected AG129 mice without treatment. Four genes, CCL2, FN1, ICAM1 and ITGB3 were significantly (*p *< 0.05) affected by the FCPLJ treatment. The CCL2 was upregulated (2.20-fold) while FN1 (− 1.66-fold), ICAM1 (− 1.74 fold) and ITGB3 (− 1.61-fold) were downregulated (Fig. 1 and Table 2) after the FCPLJ treatment. The FCPLJ treatment did not significantly affect the gene expression levels in the liver of mock infected mice (Fig. 1 and Additional file 2).

**Table 2:** Endothelial cell biology genes that were affected by the FCPLJ treatment

Gene symbol	Description	Fold regulation^a^	*p* value^b^
CCL2	Chemokine (C–C motif) ligand 2	2.20	0.025930
ITGB3	Integrin beta 3	− 1.61	0.006935
FN1	Fibronectin 1	− 1.66	0.020792
ICAM1	Intercellular adhesion molecule 1	− 1.74	0.027272

AG129 mice were intraperitoneally inoculated with 2 × 10^6^ PFU dengue virus, New Guinea C strain and orally treated with 1000 mg/kg BW FCPLJ

^a^Fold regulations of > 1.5 and > − 1.5 were considered upregulation and downregulation, respectively as compared to the gene expression level in the infected group

^b^The differences in gene expression level was considered significance at a *p* value of < 0.05

### Discussion

This study demonstrated that dengue virus infection in the liver of AG129 mice upregulated (except CASP3 and TYMP) several genes coding for the proteins involved in endothelial permeability processes. Although the viral RNA level in the liver was not affected by the FCPLJ treatment, the expression of CCL2, ICAM1, FN1 and ITGB3 genes were affected. Our previous study has also demonstrated that the FCPLJ treatment did not affect the plasma viral RNA and NS1 levels in a similar dengue mouse model [13].

During dengue virus infection, the endothelial permeability was enhanced by the interaction of dengue virus antigens and/or immune cells such as leukocytes, macrophages and platelets [18, 19]. These interactions could result in expression of cytokines and chemokines from the immune cells and endothelial cells [20]. Indeed, most of the genes that were upregulated by dengue virus infection have been reported to be involved in leukocyte-endothelial cell interaction (CCL2, SELL, SELP, SELPLG), platelet-monocyte aggregation (CCL2, PF4), endothelial cell activation and adhesion (VCAM-1, ICAM-1, ITGB3) and platelet activation (PF4) mechanisms (Additional file 3).

The gene that was mostly upregulated by dengue virus infection in the liver was serpine1 (27.9817-fold upregulated) or plasminogen activator protein type 1 gene (PAI-1), which plays a role in fibrinolysis and cell migration inhibition [21]. The increased level of serpine1 or PAI-1 was found in children with dengue hemorrhagic fever and was associated with thrombocytopenia, plasma leakage [22] and coagulation abnormalities [23].

The upregulation of BCL2L1 (caspase activation inhibitor), PROCR (antiapoptotic receptor) and downregulation of CASP3 genes showed the possible activation of anti-apoptotic mechanism during dengue virus infection in the liver. Apoptosis inhibition may be needed for efficient dengue virus propagation while its activation is induced by the host to clear the infected cells [24, 25].

The MCP-1/CCL2 gene that was upregulated by FCPLJ treatment during dengue viral infection encodes a multifunctional protein. The MCP-1 produced by dengue virus infected monocytes could increase the endothelial permeability [26]. In contrast, this protein could promote endothelial wound repair as it induced migration of human umbilical vein endothelial cell monolayers [27]. Another possible function of MCP-1 is in apoptosis as MCP-1 treated-human umbilical vein endothelial cells induced caspase-9 activation, mitochondrial alteration and p53 upregulation [28]. Furthermore, the MCP-1/CCL2 was among the upregulated genes in the peripheral blood mononuclear cells treated with *C. papaya* leaf aqueous extract in vitro [9].

The FCPLJ treatment downregulated ITGB3, ICAM1 and FN1 genes, which were upregulated during dengue virus infection. These genes are known for their roles in vascular integrity and permeability during dengue infections (Additional file 3). In detail, β1 and β3 integrins, which were surface receptors for vascular endothelial cells and platelets, were found to have a role in maintaining the capillary integrity. The ITGB3 transcript was found to be upregulated in human dermal microvascular endothelial cell line-1 infected with dengue virus [29]. Whereas, ICAM-1 as mentioned earlier, plays a role in mediating the adhesion of leukocytes or immune cells with endothelial cells to facilitate transmigration during dengue infections. The increase in ICAM-1 expression was also reported in samples of dengue virus infected mast cells [30].

In conclusion, infection of AG129 mice with non-mouse adapted dengue virus, New Guinea C strain, has caused changes in the regulation of genes associated with endothelial permeability within the liver tissue. The FCPLJ treatment has affected the regulation of four genes, CCL-2/MCP-1 (upregulated), ITGB3, ICAM1 and FN1 (downregulated), which were involved in endothelial permeability regulation during dengue virus infections. Further proteomic studies need to be conducted to functionally define the roles of the genes or proteins affected by FCPLJ treatment during dengue virus infection.

## Limitations

As our study have provided the list of endothelial cell biology related genes affected by dengue virus infection in both AG129 mice with and without FCPLJ treatment, it comes with several limitations as listed below.

1. As a preliminary study, the data was gathered from a limited number of mice per experimental group treated with a single dose of FCPLJ. Hence, the dose related effect could not be highlighted in this study.
2. The study was based on the mRNA production. Proteomic studies will further confirm the functional activities of the genes affected.
3. The dengue fever mouse model in this study was not exactly reflects the dengue hemorrhagic condition as the infection by dengue virus New Guinea C strain was less symptomatic. Hence, future studies using symptomatic dengue virus strain is crucial for the evaluation of this study particularly on the sign of plasma leakage in the infected mice.
4. The total RNA was isolated from liver tissues, which comprised of the sinusoidal endothelial and parenchymal tissues. Therefore, other possible nonspecific functions of the affected genes could not be excluded in this study.

## Additional files

**Additional file 1.** The level of viral RNA in the liver of dengue virus infected AG129 mice.

**Additional file 2.** Fold regulation of 84 genes associated with endothelial cell biology.

**Additional file 3.** The endothelial biology related genes associated with dengue infection.

## Abbreviations

BCL2L1: bcl2-like 1
BW: body weight
CASP3: caspase 3
CCL2: chemokine (C–C motif) ligand 2
Ct: cycle threshold
cDNA: complementary deoxyribonucleic acid
FCPLJ: freeze-dried *C. papaya* leaf juice
FN1: fibronectin 1
ICAM1: intercellular adhesion molecule 1
ITGB3: integrin beta 3
KIT: kit oncogene
MMP9: matrix metallopeptidase 9
PFU: pore forming unit
PF4: platelet factor 4
PROCR: protein C receptor, endothelial
qRT-PCR: quantitative reverse transcription-polymerase chain reaction
RNA: ribonucleic acid
SELL: selectin, lymphocyte
SELPLG: selectin, platelet (p-selectin) ligand
SELP: selectin, platelet
SERPINE1: serine (or cysteine) peptidase inhibitor, clade E, member 1
TGFB1: transforming growth factor, beta 1
TYMP: thymidine phosphorylase
VCAM1: vascular cell adhesion molecule 1
