# Supplementary figures and images for: Preliminary study on the expression of endothelial cell biology related genes in the liver of dengue virus infected mice treated with Carica papaya leaf juice
Source: BMC Res Notes. 2019 Apr 3;12:206. doi: 10.1186/s13104-019-4242-z (PMC6448258; doi:10.1186/s13104-019-4242-z)

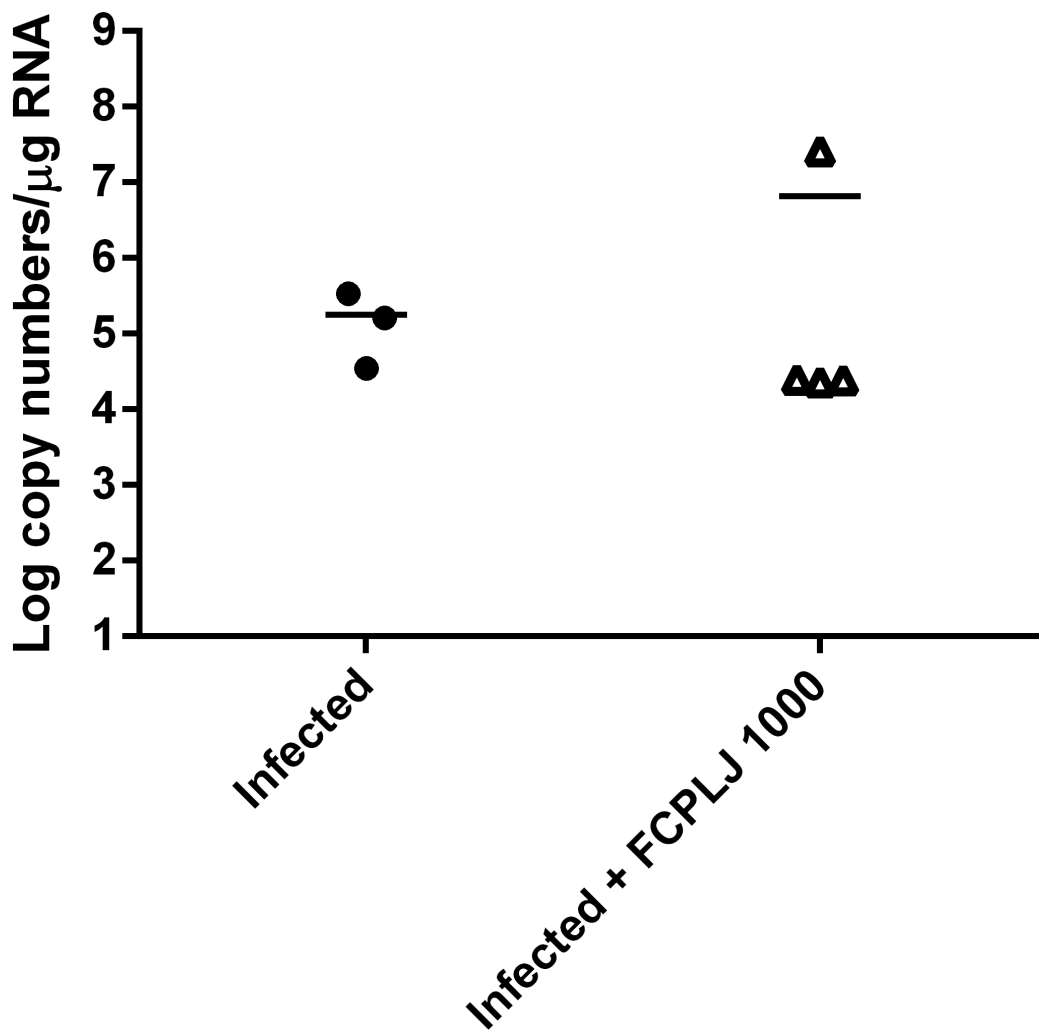

Additional file 1. The level of viral RNA in the liver of dengue virus infected AG129 mice.

Supplement: Supplementary file 1 — Additional file 1. The level of viral RNA in the liver of dengue virus infected AG129 mice. [file 13104_2019_4242_MOESM1_ESM.pdf]
