# Supplementary material for: Preliminary study on the expression of endothelial cell biology related genes in the liver of dengue virus infected mice treated with Carica papaya leaf juice
Source: BMC Res Notes. 2019 Apr 3;12:206. doi: 10.1186/s13104-019-4242-z (PMC6448258; doi:10.1186/s13104-019-4242-z)
Supplement: Supplementary file 2 — Additional file 2. Fold regulation of 84 genes associated with endothelial cell biology. [file 13104_2019_4242_MOESM2_ESM.pdf]

Additional file 2. Fold regulations of 84 genes associated with endothelial cell biology.

Gene name	Gene symbol	Up-Down Regulation (comparing to mock infected mice)					
		Mock + FCPJ		Infected		Infected + FCPLJ	
		Fold Regulation	P value	Fold Regulation	P value	Fold Regulation	P value
Angiotensin I converting enzyme (peptidyl-dipeptidase A) 1	Ace	1.3119	0.912563	3.7109	0.101063	3.1479	0.10836
A disintegrin and metallopeptidase domain 17	Adam17	-1.2957	0.467052	-1.139	0.173596	-1.2118	0.371617
Angiotensinogen (serpin peptidase inhibitor, clade A, member 8)	Agt	-3.0592	0.20179	-1.2972	0.806737	-1.1961	0.950261
Angiotensin II receptor, type 1a	Agtr1a	-1.4092	0.168223	-1.2699	0.197983	-1.0209	0.888696
Angiopoietin 1	Angpt1	3.1867	0.365783	1.3415	0.449829	1.5422	0.230932
Annexin A5	Anxa5	1.1196	0.634213	1.0653	0.887241	1.0575	0.907425
Apolipoprotein E	Apoe	-2.2038	0.128887	-1.7258	0.072772	-1.5202	0.311455
Bcl2-associated X protein	Bax	1.5148	0.366311	1.2537	0.192288	1.1122	0.409271
B-cell leukemia/lymphoma 2	Bcl2	-1.3265	0.289674	1.563	0.362269	1.2307	0.566874
Bcl2-like 1	Bcl2l1	1.3313	0.391199	**2.3017**	**0.005111**	**1.6367**	**0.032868**
Caspase 1	Casp1	1.4148	0.471517	1.6234	0.262211	1.6425	0.123158
Caspase 3	Casp3	-1.0772	0.764188	**-1.5606**	**0.000338**	**-1.7293**	**0.006436**
Caveolin 1, caveolae protein	Cav1	1.6119	0.438624	-1.255	0.655535	-1.1135	0.536161
Chemokine (C-C motif) ligand 2	Ccl2	-1.3017	0.858253	**17.9509**	**0.010773**	**39.4756**	**0.001338**
Chemokine (C-C motif) ligand 5	Ccl5	1.0944	0.723954	1.5672	0.202747	2.1223	0.135846
Cadherin 5	Cdh5	-1.6267	0.633285	1.4654	0.094229	**1.5709**	**0.019515**

CASP8 and FADD-like apoptosis regulator	Cflar	1.2553	0.609846	1.0511	0.981578	-1.3288	0.275595
Collagen, type XVIII, alpha 1	Col18a1	-2.2974	0.096028	-1.7121	0.0688	-1.7515	0.054778
CASP2 and RIPK1 domain containing adaptor with death domain	Cradd	-2.1122	0.108557	-1.4967	0.04209	-1.3174	0.453895
Chemokine (C-X3-C motif) ligand 1	Cx3cl1	1.915	0.389442	1.2079	0.481899	1.6506	0.465245
Chemokine (C-X-C motif) ligand 1	Cxcl1	-2.4066	0.449814	96.7451	0.226103	27.6943	0.152266
Chemokine (C-X-C motif) ligand 2	Cxcl2	1.0907	0.666302	4.4172	0.101026	3.8194	0.053476
Chemokine (C-X-C motif) receptor 5	Cxcr5	1.0532	0.595396	2.0658	0.407763	1.8259	0.433751
Endothelin 1	Edn1	1.0134	0.785468	1.5762	0.120124	1.3432	0.298466
Endothelin 2	Edn2	2.8556	0.380402	3.2067	0.381623	3.1751	0.426554
Endothelin receptor type A	Ednra	-1.6198	0.433285	-1.0486	0.807729	-1.1163	0.802287
Endoglin	Eng	-1.1918	0.276359	-1.2546	0.200544	-1.1945	0.245054
Coagulation factor II (thrombin) receptor	F2r	1.1202	0.653099	-1.3979	0.17736	-1.0482	0.663244
Coagulation factor II (thrombin) receptor-like 1	F2rl1	1.2191	0.490842	1.5635	0.44033	1.6202	0.39282
Coagulation factor III	F3	-1.5699	0.363344	-1.2803	0.450459	-1.6413	0.168523
Fas (TNF receptor superfamily member 6)	Fas	1.0227	0.640562	1.7316	0.0611	1.4664	0.01789
Fas ligand (TNF superfamily, member 6)	Fasl	3.4835	0.365925	4.1527	0.336263	4.9536	0.259677
Fibroblast growth factor 1	Fgf1	1.2779	0.514682	-1.0532	0.729299	1.0438	0.98369
Fibroblast growth factor 2	Fgf2	-1.4667	0.519279	1.5548	0.504426	1.322	0.613223
FMS-like tyrosine kinase 1	Flt1	-1.3428	0.476342	-1.1075	0.634909	-1.0778	0.584916
Fibronectin 1	Fn1	-1.0555	0.940402	**1.4884**	**0.038106**	-1.1149	0.647467
Hypoxia inducible factor 1, alpha subunit	Hif1a	1.1418	0.644051	1.0264	0.983842	-1.2901	0.201037

Intercellular adhesion molecule 1	Icam1	-1.1125	0.691758	**4.9573**	**0.001673**	**2.8445**	**0.013989**
Interleukin 11	Il11	3.3737	0.363725	3.1797	0.374698	3.4084	0.404424
Interleukin 1 beta	Il1b	-1.9781	0.148805	-1.0025	0.821915	1.6628	0.198637
Interleukin 3	Il3	1.9288	0.506878	2.7338	0.412906	4.9258	0.38724
Interleukin 6	Il6	1.7145	0.422766	4.0619	0.16246	8.1486	0.236125
Interleukin 7	Il7	-1.0111	0.785749	1.9848	0.277431	2.376	0.129294
Integrin alpha 5 (fibronectin receptor alpha)	Itga5	-1.2652	0.495737	-1.6885	0.197128	-1.5895	0.180461
Integrin alpha V	Itgav	-1.0259	0.818743	-1.0044	0.872508	-1.2456	0.234202
Integrin beta 1 (fibronectin receptor beta)	Itgb1	-1.0443	0.902535	-1.0085	0.873437	-1.2784	0.077281
Integrin beta 3	Itgb3	1.2435	0.47616	**2.3885**	**0.004266**	1.4858	0.044112
Kinase insert domain protein receptor	Kdr	-1.335	0.197805	-1.2254	0.210866	-1.3634	0.169328
Kit oncogene	Kit	-1.1109	0.881768	**1.51**	**0.019643**	1.4393	0.055685
Matrix metallopeptidase 1a (interstitial collagenase)	Mmp1a	-2.3237	0.221962	-3.4647	0.053504	**-2.8722**	**0.03194**
Matrix metallopeptidase 2	Mmp2	-1.5215	0.528847	1.0829	0.615787	-1.5134	0.774342
Matrix metallopeptidase 9	Mmp9	1.2445	0.518885	**4.2087**	**0.017157**	**5.2843**	**0.01207**
Nitric oxide synthase 3, endothelial cell	Nos3	1.5533	0.215033	1.847	0.108387	1.43	0.363302
Natriuretic peptide type B	Nppb	3.7538	0.370437	2.7871	0.385631	3.8616	0.392298
Natriuretic peptide receptor 1	Npr1	-1.6868	0.203855	-1.1408	0.470584	-1.2494	0.501203
Occludin	Ocln	1.0306	0.932963	-1.9027	0.252598	-1.4541	0.321551
Platelet derived growth factor receptor, alpha polypeptide	Pdgfra	-1.2547	0.85405	1.3838	0.088396	-1.2074	0.579715
Platelet/endothelial cell adhesion molecule 1	Pecam1	1.1963	0.594585	1.3343	0.385076	1.2712	0.304556

Platelet factor 4	Pf4	1.5189	0.197	**2.8456**	**0.002018**	3.3073	0.060086
Placental growth factor	Pgf	1.4273	0.512546	3.7744	0.368246	1.5017	0.511236
Plasminogen activator, tissue	Plat	1.9186	0.399209	2.6508	0.159437	2.1783	0.114067
Plasminogen activator, urokinase	Plau	1.1912	0.399823	-1.325	0.88663	1.2591	0.461192
Plasminogen	Plg	-1.1827	0.273182	1.0209	0.920809	1.0165	0.910078
Protein C receptor, endothelial	Procr	1.239	0.35026	**2.6883**	**0.010671**	2.0945	0.118497
Prostaglandin I2 (prostacyclin) synthase	Ptgis	2.0294	0.220971	2.3739	0.169568	1.818	0.091359
Prostaglandin-endoperoxide synthase 2	Ptgs2	3.1416	0.399059	2.3481	0.475332	4.6897	0.274944
Selectin, endothelial cell	Sele	1.6203	0.474517	10.3567	0.141789	6.599	0.155466
Selectin, lymphocyte	Sell	-1.3197	0.29598	**2.9629**	**0.008503**	2.3749	0.158567
Selectin, platelet	Selp	1.4846	0.428479	**1.7322**	**0.00361**	1.4627	0.043283
Selectin, platelet (p-selectin) ligand	Selplg	1.2857	0.514328	**4.0692**	**0.012107**	**3.9683**	**0.012153**
Serine (or cysteine) peptidase inhibitor, clade E, member 1	Serpine1	2.3574	0.183933	**27.9817**	**0.046792**	**19.5595**	**0.046917**
Superoxide dismutase 1, soluble	Sod1	1.5566	0.208119	1.2105	0.25248	1.0066	0.975448
Endothelial-specific receptor tyrosine kinase	Tek	1.1191	0.648282	-1.1037	0.636718	-1.0845	0.539672
Tissue factor pathway inhibitor	Tfpi	2.0256	0.247811	-1.4881	0.141154	-1.1425	0.640883
Transforming growth factor, beta 1	Tgfb1	1.1833	0.570468	**1.9354**	**0.018087**	**1.8707**	**0.011561**
Thrombomodulin	Thbd	3.2334	0.3033	7.4205	0.079645	**5.3421**	**0.001708**
Thrombospondin 1	Thbs1	-1.5869	0.425617	1.0457	0.789169	-1.1991	0.660612
Tissue inhibitor of metalloproteinase 1	Timp1	2.418	0.279454	9.844	0.061165	**4.7054**	**0.028524**
Tumor necrosis factor	Tnf	2.0891	0.470058	6.7303	0.36329	6.565	0.402977

Tumor necrosis factor (ligand) superfamily, member 10	Tnfsf10	1.0073	0.699918	-1.611	0.096423	-1.0014	0.886055
Thymidine phosphorylase	Tymp	-1.9445	0.11455	**-2.1416**	**0.015268**	**-1.6342**	**0.038718**
Vascular cell adhesion molecule 1	Vcam1	2.3588	0.212504	**6.4031**	**0.039317**	**3.9085**	**0.001934**
Vascular endothelial growth factor A	Vegfa	-1.1051	0.763052	-1.4936	0.131162	-1.5041	0.07973
Von Willebrand factor homolog	Vwf	-1.3799	0.261699	1.2601	0.477788	1.0608	0.631913
