# Supplementary material for: Preliminary study on the expression of endothelial cell biology related genes in the liver of dengue virus infected mice treated with Carica papaya leaf juice
Source: BMC Res Notes. 2019 Apr 3;12:206. doi: 10.1186/s13104-019-4242-z (PMC6448258; doi:10.1186/s13104-019-4242-z)
Supplement: Supplementary file 3 — Additional file 3. The endothelial biology related genes associated with dengue infection. [file 13104_2019_4242_MOESM3_ESM.pdf]

Additional file 3. The endothelial cell biology related genes associated with dengue infection.

Description (Gene symbol)	Functions and association with dengue infection
Bcl2-like 1 (BCL2L1)	Encodes a member of the BCL-2 family of apoptosis regulators which may be important during dengue virus induced apoptosis (1).
Caspase 3 (CASP3)	Encodes a proapoptotic protein. Cytophatic effect (CPE) in dengue virus infected cells was caused by the induction of caspase 3 dependent apoptosis (2).
Chemokine (C-C motif) ligand 2 (CCL2)	Encodes a monocyte chemoattractant protein 1 (MCP-1) which function in monocyte trafficking across the endothelial barriers (3).<br><br>The CCL2 was high in the plasma of dengue patients could be due to the platelet-monocyte aggregation (4).
Intercellular adhesion molecule 1 (ICAM1)	Encodes ICAM-1 proteins during endothelial cell activation (5).<br><br>Dengue virus stimulates monocytes or endothelial cell to express ICAM-1 which may facilitate inflammation or coagulation processes (6).
Integrin beta 3 (ITGB3)	Expressed on activated endothelial cells and important adhesion molecules participated in anti-DENV NS1 antibody-mediated platelet phagocytosis (7).<br><br>It was also required for dengue virus entry into the endothelial cells (8).
Matrix metallopeptidase 9 (MMP9)	Enhances endothelial permeability during dengue virus infection *in vitro* (9).
Platelet factor 4 (PF4)	Produced by the activated platelet during dengue infection which possibly involve in platelet-monocyte aggregation at the endothelium (10, 11).
Protein C receptor, endothelial (PROCR)	Functions in regulation of thrombin generation, anti-inflammatory, antiapoptotic and barrier protection by the association with activated protein C (APC) which was downregulated during dengue virus infection (12).
Selectin, lymphocyte (SELL)	Encode for L-selectin which important in leukocyte tethering or rolling in leukocyte-endothelial cell interactions (5).
Selectin, platelet (SELP)	Encode for P-selectin which important in leukocyte tethering or rolling in leukocyte-endothelial cell interactions (5).

	Interaction of platelets and monocyte through P-selectin can lead to the secretion of cytokines (4).
Selectin, platelet (p-selectin) ligand (SELPLG)	Encode for P-selectin ligand or P-selection glycoprotein ligand 1 (PSLG-1) (5) which is important in leukocyte tethering and platelet-endothelial interaction which leads to neutrophils and monocytes recruitment (13).
Serine (or cysteine) peptidase inhibitor, clade E, member 1 (SERPINE1)	Also known as plasminogen activator inhibitor-1 and its production is modulated by the transforming growth factor beta-1 cytokine (5).<br><br>The level of PAI-1 was increased in the plasma of children with severe dengue and associated with platelet counts and plasma leakage (14).
Transforming growth factor, beta 1 (TGFB1)	A multifunctional cytokine. It was detected high in the sera and peripheral blood mononuclear cells of dengue hemorrhagic fever patients (15).
Vascular cell adhesion molecule 1 (VCAM1)	The expression of VCAM-1increased in activated microvascular endothelial cells after exposure to culture supernatants derived from dengue virus infected monocytes (16).

References

1. Su HL, Lin YL, Yu HP, Tsao CH, Chen LK, Liu YT, et al. The effect of human bcl-2 and bcl-X genes on dengue virus-induced apoptosis in cultured cells. Virology. 2001;282(1):141-53.
2. Liu Y, Liu H, Zou J, Zhang B, Yuan Z. Dengue virus subgenomic RNA induces apoptosis through the Bcl-2-mediated PI3k/Akt signaling pathway. Virology. 2014;448:15-25.
3. Shi C, Pamer EG. Monocyte recruitment during infection and inflammation. Nat Rev Immunol. 2011;11(11):762-74.
4. Hottz ED, Medeiros-de-Moraes IM, Vieira-de-Abreu A, de Assis EF, Vals-de-Souza R, Castro-Faria-Neto HC, et al. Platelet activation and apoptosis modulate monocyte inflammatory responses in dengue. J Immunol. 2014;193(4):1864-72.
5. The Universal Protein Resource [Internet]. 2018 [cited 7/2/2018]. Available from: http://www.uniprot.org/.
6. Yeh TM, Liu SH, Lin KC, Kuo C, Kuo SY, Huang TY, et al. Dengue virus enhances thrombomodulin and ICAM-1 expression through the macrophage migration inhibitory factor induction of the MAPK and PI3K signaling pathways. PLoS One. 2013;8(1):e55018.
7. Wan SW, Yang YW, Chu YT, Lin CF, Chang CP, Yeh TM, et al. Anti-dengue virus nonstructural protein 1 antibodies contribute to platelet phagocytosis by macrophages. Thromb Haemost. 2016;115(3):646-56.
8. Zhang JL, Wang JL, Gao N, Chen ZT, Tian YP, An J. Up-regulated expression of beta3 integrin induced by dengue virus serotype 2 infection associated with virus entry into human dermal microvascular endothelial cells. Biochem Biophys Res Commun. 2007;356(3):763-8.

9. Luplertlop N, Misse D, Bray D, Deleuze V, Gonzalez JP, Leardkamolkarn V, et al. Dengue-virus-infected dendritic cells trigger vascular leakage through metalloproteinase overproduction. EMBO Rep. 2006;7(11):1176-81.
10. Srichaikul T, Nimmannitya S, Sripaisarn T, Kamolsilpa M, Pulgate C. Platelet function during the acute phase of dengue hemorrhagic fever. The Southeast Asian journal of tropical medicine and public health. 1989;20(1):19-25.
11. Trugilho MRO, Hottz ED, Brunoro GVF, Teixeira-Ferreira A, Carvalho PC, Salazar GA, et al. Platelet proteome reveals novel pathways of platelet activation and platelet-mediated immunoregulation in dengue. PLoS Pathog. 2017;13(5):e1006385.
12. Cabello-Gutierrez C, Manjarrez-Zavala ME, Huerta-Zepeda A, Cime-Castillo J, Monroy-Martinez V, Correa BB, et al. Modification of the cytoprotective protein C pathway during Dengue virus infection of human endothelial vascular cells. Thromb Haemost. 2009;101(5):916-28.
13. Shigeta A, Matsumoto M, Tedder TF, Lowe JB, Miyasaka M, Hirata T. An L-selectin ligand distinct from P-selectin glycoprotein ligand-1 is expressed on endothelial cells and promotes neutrophil rolling in inflammation. Blood. 2008;112(13):4915-23.
14. Djamiatun K, Faradz SM, Setiati TE, Netea MG, van der Ven AJ, Dolmans WM. Increase of plasminogen activator inhibitor-1 and decrease of transforming growth factor-b1 in children with dengue haemorrhagic fever in Indonesia. J Trop Pediatr. 2011;57(6):424-32.
15. Agarwal R, Elbishbishi EA, Chaturvedi UC, Nagar R, Mustafa AS. Profile of transforming growth factor-beta 1 in patients with dengue haemorrhagic fever. Int J Exp Pathol. 1999;80(3):143-9.
16. Kelley JF, Kaufusi PH, Nerurkar VR. Dengue hemorrhagic fever-associated immunomediators induced via maturation of dengue virus nonstructural 4B protein in monocytes modulate endothelial cell adhesion molecules and human microvascular endothelial cells permeability. Virology. 2012;422(2):326-37.
